# Supplementary material for: Patterns of Intron Gain and Loss in Fungi
Source: PLoS Biol. 2004 Nov 30;2(12):e422. doi: 10.1371/journal.pbio.0020422 (PMC532390; doi:10.1371/journal.pbio.0020422)
Supplement: Table S1 — Also available at http://genes.mit.edu/NielsenEtAl/. (4.3 MB ZIP). [file pbio.0020422.st001.zip › NielsenEtAl/html/597.html]

AN3702.1.NCU09463.1.MG02432.1.FG06009.1


```
 CLUSTAL W (1.82) Multiple Sequence Alignments - Introns Inserted


Sequence 1: NCU09463.1	1123 aa
Sequence 2: MG02432.1	1125 aa
Sequence 3: FG06009.1	1116 aa
Sequence 4: AN3702.1	1062 aa
Alignment Length: 1138 aa
Number Identitical Residues: 521 aa
Alignment Score (without introns) 27622


MG02432.1 	MAAAATPIPTMDGLQIS--KTKELKG0TEKRTALVAIEKKYQQKWEEDGVFNFNAPSLDE
NCU09463.1	MADTAAVAKGVENLSVSASKTKELKG~TEKRDTLIEIEKRYQQKWEQEGVFEVDAPSTAE
FG06009.1 	MASTDAVPKAMESLHIS--KTKELKG0TEKRDTLIAIEKKYQKIWEDQHVFEADSPSTTE
AN3702.1  	--------------------------~---------------------------------
          	                                                            

MG02432.1 	YPLDSISPEELRTKFPKFFGTIAYPYMNGVLHIGHYFTMSKVEFQMGFSRMQGKRTFWPQ
NCU09463.1	FPLDAITPDELRQKHPKFFGTIAYPYMNGRLHAGHAFSFSKIEYHTGFARMQGKRALFPQ
FG06009.1 	VPLHSVSAAELREQQPKFFGCMAYPYMNGTLHAGHSFSVSKVEFAAGVARMQGKRTLFPM
AN3702.1  	-----MTPDELREKYPKFFGTMAYPYMNGTLHAGHSFTASKVEFMAATARMEGKRALFPL
          	     ::. *** : ***** :******* ** ** *: **:*:  . :**:***:::* 

MG02432.1 	GYHCTGLPIKACADKLAEEVKKFGKEFDGYNEESEEPEAAPAPKPTAGQREDLTKFTATK
NCU09463.1	GYHCTGLPIKASADKLVKEIEMFGQEFERYKEDEVVEGAAPAAAAAPKTKEDLTKFNAKK
FG06009.1 	GFHCTGMPIKACADKLVNEVKKFGQDFSGYKEEE---EAVVEEAPKAQTKEDITKFKATK
AN3702.1  	GFHCTGMPIKACADKLADEVKKFGKNFEGYKDEDE-ETAAVAAPTQEVKAEQQEKFSGKK
          	*:****:****.****..*:: **::*. *:::.    *.    .     *:  **...*

MG02432.1 	GKANAKTVKKK~YQFQIMESIGIPRQEIHRFADSQYWLDYFPPLCHEHLKATGARIDWRR
NCU09463.1	GKTVAKTGGAK~YQFQILKSLGIPVSEIHKFADPQYWLHYFPPECKKDLTNFGARIDWRR
FG06009.1 	GKAAAKTVKMK2YQFQIMQAIGISTEEIHKFADPQYWLQHFPPLCREDLTNFGCRIDWRR
AN3702.1  	SKAAAKTVKMK~YQFQIMLAIGIPIEEIHKFADASYWLQHFPPLAIRDLDSLGAGIDWRR
          	.*: ***   * *****: ::**. .***:***..***.:*** . ..*   *. *****

MG02432.1 	QFVTTDANPYYDSFVRWTMNRLREMNKVKFGKRYTIYSAKDGQPCMDHDRGEGEGVGPQE
NCU09463.1	QFVTTDANPYYDAFVRWQMNRLLELNKIKFGKRYTIYSIKDGQPCMDHDRSEGEGVLPQE
FG06009.1 	SFVTTDANPYYDAFVRWQMNRLKELNKIKFGKRYTIYSIKDGQPCMDHDRSDGEGAGPQE
AN3702.1  	QMVTTDANPYYDAFVRWQMNRLYELGKIQYGNRYTIYSPKDGQPCMDHDRTEGEGIGPQE
          	.:**********:**** **** *:.*:::*:****** *********** :***  ***

MG02432.1 	YTALKLKVVEWAPKAAAALEGKVPSDVSVFLVPATLRPETMYGQTCCFVGPKLKYGLFKA
NCU09463.1	YTALKLKVTEWAPKAAEALKGKLPEGANVYLCPATLRPETMYGQVCCFVGPALKYGVFKA
FG06009.1 	YTALKLKVLEWAPKAAEALKGKLPEQANVYCVPATLRPETMYGQTCCFVGPKITYGIFKV
AN3702.1  	YTAMKLKVKEWAPKIAELVKGKIEDDADVYFIPATLRPETMYGQNCCFLGPKIEYGIFRV
          	***:**** ***** *  ::**: . ..*:  ************ ***:** : **:*:.

MG02432.1 	SEKEYFVITERSAKNMAFQGLLEKDMAIPEKLADLV------GADIVGTRVNAPLSVHKE
NCU09463.1	AENEYFVITERAAKNMAYQGIFEKEGVI-EKAADIV------GSDLIGTLVNAPLSVHKE
FG06009.1 	NETDYYVMTERAARNMAYQGIFAKEGVI-EQTAEVG------GADLVGTLVDAPLSLHKE
AN3702.1  	KEKQYYICTKRGAWNMAFQGTFFDSEHFPKTQDELPTVLTAPGSAFVGTLVDAPLSFHKD
          	 *.:*:: *:*.* ***:** : ..  :.:   :: :  ::.*: ::** *:****.**:

MG02432.1 	-VRILPMETVKETKGTGVVTSVPSDSPDDYATVMDLAKKADYYGITKEWAELEIVPIIDT
NCU09463.1	-VYVLPMDTVLATKGTGVVTSVPSDSPDDCAMMTELAKKPEFYGIQKEWAEKEIVSVIKT
FG06009.1 	GVRILPMETVLPTKGTGVVTSVPSDSPDDYATVTDLAKKADYYGITKEWAELEIFPIIET
AN3702.1  	GVRILPMEGVSATKGTGVVTCVPSDSPDDYATLLDLAKKPEYYGIQKEWAELEIFPLIET
          	.* :***: *  ********.******** * : :****.::*** ***** **..:*.*

MG02432.1 	PSYGDLCAKFLVEKLKIASPKDVKQLTEAKELAYKEGYYQGTMKIGKYAGEKVEAAKPKV
NCU09463.1	PTS-DLLAPYLVKKLKINSPKDAKQLLEAKELAYKEGFYQGIMNYGDFKGEKVETAKPKV
FG06009.1 	PSYGDLCAPFLVKKLKIASPKDTKQLEEAKELAYKEGFYQGVLKVGEFKGEKVEVAKPKV
AN3702.1  	PTYGNLTAPALVKKLKINSPKDVNQLAQAKELAYGEAFYKGTMIVGEFKGEPVSAAKDKI
          	*: .:* *  **:**** ****.:** :****** *.:*:* :  *.: ** *..** *:

MG02432.1 	RQDLINESLAFAYAEPENKVVSRSGDECIVALLDQWYLDYGEE--KWRDQALEFVENADG
NCU09463.1	RQQLIDAGDAFAYSEPENKVVSRSGDECSVALMDQWYIDYGED--SWRTVLYDYVENKDG
FG06009.1 	RQQMIDAGQAFAYSEPERKVTSRSGDDCIVSLMDQWYLDYGEE--SWKKTTLDWVEN---
AN3702.1  	RKALYDSGDAFPFADPMGKVVSRSGDDCVVAYLGQWFLNYGENDAKWQKDTLDHVVN---
          	*: : : . **.:::*  **.*****:* *: :.**:::***:.:.*:    : * *   

MG02432.1 	NGLETYTTETKHAFKGMLNWLNQWACARTYGLGSKLPWDPKFLVESLSDSTVYMAYYTQV
NCU09463.1	KGINTYYADTQHAFKGVIGWLKQWACARTYGLGSKLPWDPNFLVESLSDSTVYMAYYTVA
FG06009.1 	-GLNTYTAETKNQFEGVLNWLNQWACARSFGLGSKLPWDPQFLVESLSDSTIYMAYYTIA
AN3702.1  	-TLNTYSNETKNGFEKNLSWLNRWACARTYGLGSKLPWDPQFLVESLSDSTVYMAYYTIA
          	  ::**  :*:: *:  :.**::*****::**********:**********:****** .

MG02432.1 	PFLHKDNFGRVRGSADIGPEQMLDEVWDYIFCRTEMTDKLVEDSKIPKTTLEAMRREFQY
NCU09463.1	HWLHRDLFGREKGKGNIGADQMIDEVWDYIFCRTELSDHLVTKSGIPKETLDSMRREFQY
FG06009.1 	HYLHNDIFGKTKGLANIGPEAMTDEVWDYVFCRGELTDEVLN-SKIPKDTLEKMRREFEY
AN3702.1  	HLLHGDRYGKTTGKLNIKAEQMIDEVWDYVFCRREISDELISKSGLSKDALQAMRREFEY
          	  ** * :*:  *  :* .: * ******:*** *::*.:: .* :.* :*: *****:*

MG02432.1 	SYPLDLRVSGK0DL~IGNHLTFAIYCHIALFPREYWPKSFRVNGHLMMNGEKMSKSTGNF
NCU09463.1	FYPLDIRVSGK~DL~IPNHLTFWLYNHIALFPREYWPKSVRANGHLQLNGEKMSKSTGNF
FG06009.1 	FYPLDVRVSGK~DL0IPNHLSMHLYCHTALFPREYWPRSIRANGHLMLNGEKMSKSTGNF
AN3702.1  	WYPMDVRVSGK~DL~IQNHLTFFLYIHVALFPPQYWPRGVRANGHLLLNGDKMSKSTGNF
          	 **:*:***** ** * ***:: :* * **** :***:..*.**** :**:*********

MG02432.1 	MTLDEATRKYGADALRMAIADAGDGVNDANFDEDVADNTILRLFTTREWIEDAVKNIGEL
NCU09463.1	MTLDDVVKKYGADAARVALADAGDGISDSNFVEDVADNTILRFYTNKEWIEETLKDE-SL
FG06009.1 	MTLRDLTQKYGADASRIALADAGDGVNDANFEEDVADTNILRLYTLKEWCEEMVQDQDQL
AN3702.1  	LTLKDSVDKFGADATRIAFADAGDGIEDANFEESVANSNILRLFTLKEWIEEVVKDE-SL
          	:** : . *:**** *:*:******:.*:** *.**:..***::* :** *: :::  .*

MG02432.1 	RTGELNDFQDALFTNELNMLVRESERHYKD~TAYKLALQSAWYNFSSAISFYRESCAAAG
NCU09463.1	RTGELNSFQDALFDNEMNALVNEARKHYEE~TSYKLALKAAHYDFLNARDMYREACAAAG
FG06009.1 	RTGEFNSFQDALFINDINAVTKEAVEQYVN~TNYKLALKAGLYELTSARDFYREACAAAN
AN3702.1  	RTGPADHFWDKVFDNEINTLVREGKKNYQD2TNFKLALKSSLYDLVGARDAYREACISAG
          	***  : * * :* *::* :..*. .:* : * :****::. *:: .* . ***:* :*.

MG02432.1 	LKLHRDVVLRYVEMQ~ALVLLPVAPHWAEYIWLEVLGK~KETIQHARWPEVPAVDASLAA
NCU09463.1	IPLHKDLVTKYIRLQ0ALVITPIAPHWADYVWQECLGE~PKSIQFARWPEVPAANPALTA
FG06009.1 	IKMHKDVVLRYIEVQ~TLLLAVFAPHWSEYIWLEVLKK~EGTIHNARFPEIQEVDASLSA
AN3702.1  	IGMHRDVVLRYIELQ~ALMMSPIAPHWSEYIWLEILKK0PDTIHRALFPEVAEPSPELSA
          	: :*:*:* :*:.:* :*::  .****::*:* * * :   :*: * :**:   .. *:*

MG02432.1 	AREYVKGTASNVNSAESAQLKKKAKGKEVSFDPKRPKKLTIFTAEAFPAWQQKYIDLVRE
NCU09463.1	ARDYVRTTSSAINSAEAAQLKKMAKGRQSDFDPKKPKKLTIFATENFPTWQAKYIDLLSE
FG06009.1 	KRDYVRNTASNINSAESHQLKKKAKGKETSFDPKKPKKLTIFVADKFPAWQAKYIDLLKE
AN3702.1  	ATSYVRATASSILSAEANFVKKLAKGKSAHFDPRKPKKITIFAAKKFPSWQEKYIDLVRE
          	  .**: *:* : ***:  :** ***:.  ***::***:***.:. **:** *****: *

MG02432.1 	NWDAEKNAIK-DDKELSARIGKMGEMKKAMPFVHNLKKRLQSGEDS-STVLERKLAFDEQ
NCU09463.1	VWDAATGTQKIDDKELNGRIAKMGEMKKAMPFVQALKKRLKDGEPA-EQILSRKLSFDEK
FG06009.1 	MWNTETKSVN--DKELNGKIGKMGEMKKAMPFVQNLKRRLQAGEPA-SAVLEQKLAFDEK
AN3702.1  	AFDAVSLTIN--DKELNAKVGKLGEMKKAMPFVQGLKKRLISTKEAPEIVFERKLPFDEF
          	 ::: . : :  ****..::.*:**********: **:**   : :.. ::.:**.*** 

MG02432.1 	KTLLAMVAGLKRSAGCVAVDVVAVAEGSKKGKLLTDGGKEVDVTAQAADGAVPGQPTFYF
NCU09463.1	ATLLAMIPGLKRTAGLESVQVVLVEEGSKTGKDLTNGGAEIEVTAPMAEAALPGQPSFFF
FG06009.1 	ETLQQMVPGLKRTGGLAVCDVIAVEQGTQKGVSLTDGGKEVDISAPVAEHAVPGQPTFYF
AN3702.1  	GVLKEMTVNLKKTTGAKEIEIVAVDEGGKTGEVLGSGEK---REGLQAENAVPGQPTFLF
          	 .*  *  .**:: *    ::: * :* :.*  * .*       .  *: *:****:* *

MG02432.1 	ENVEG
NCU09463.1	TNV--
FG06009.1 	ENVEA
AN3702.1  	ANIE-
          	 *:.
```
